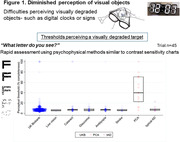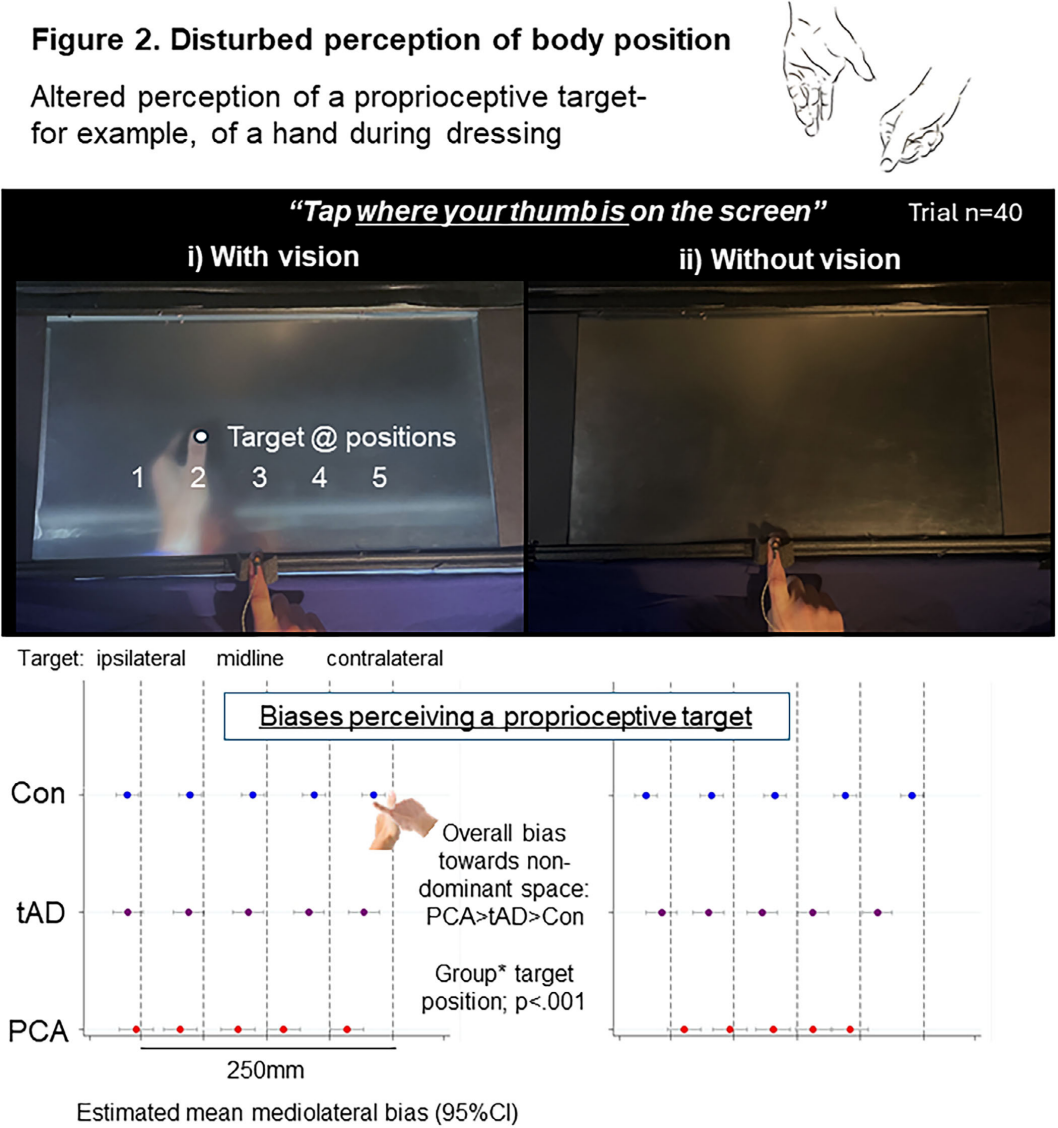# Visual and body perceptual disturbances in posterior cortical atrophy and Alzheimer’s disease

**DOI:** 10.1002/alz.083974

**Published:** 2025-01-09

**Authors:** Keir X X Yong, Yuhan Bai, Matthew J Bancroft, Axel Petzold, Paul J Foster, Diego Kaski, Juanita Hoe, Laure Pisella, John A Greenwood, Sebastian J Crutch, Brian L Day

**Affiliations:** ^1^ Dementia Research Centre, UCL Queen Square Institute of Neurology, London UK; ^2^ University College London, London UK; ^3^ Centre for Vestibular and Behavioural Neurosciences, UCL Queen Square Institute of Neurology, London UK; ^4^ NIHR Biomedical Research Centre, Moorfields Eye Hospital and UCL Institute of Ophthalmology, London UK; ^5^ University of West London, London UK; ^6^ UCBL, CNRS, INSERM, Lyon France; ^7^ UCL Experimental Psychology, London, London UK; ^8^ Whole‐Body Sensorimotor Lab, UCL Queen Square Institute of Neurology, London UK

## Abstract

**Background:**

Posterior cortical atrophy (PCA) is often considered the most common atypical Alzheimer’s disease phenotype, being characterized by progressive loss of visual and other posterior cortical functions. Early reading and other visuoperceptual difficulties prompt PCA patients presenting to eye clinics and receiving ocular misdiagnoses. Patients also report altered perception of body position‐ for example, difficulty locating ones’ arm during dressing. Disabling perceptual disturbances emerge later in typical Alzheimer’s disease (tAD) yet are poorly assessed or understood.

This project investigates visual and proprioceptive disturbances in PCA and tAD and underlying mechanisms.

**Method:**

Participants underwent psychophysical testing of vision and body perception (UCL: PCA n=28; tAD n=16; Control n=19; UKBiobank n=1,951). To evaluate perception of a visual target, participants repeatedly identified letters under increasing degradation, or incompleteness (trial n=45). To evaluate perception of a proprioceptive target, participants repeatedly indicated the position of their non‐dominant thumb without and with vision (trial n=40). Perceptual error and thresholds were determined alongside primary sensory and visual functions (vibrotactile sensitivity, acuity).

**Result:**

Thresholds for perceiving a visually degraded target were elevated in PCA compared to UKBiobank participants without or with documented eye conditions (Figure 1), consistent with higher‐order visual dysfunction (PCA sensitivity: 87%; specificity vs eye conditions: 91%).

Across groups, perceived location of a proprioceptive target (non‐dominant thumb) was shifted, or ‘biased’, towards non‐dominant space, consistent with previous work in healthy participants. However, proprioceptive biases were considerably elevated in both patient relative to control groups, particularly with targets positioned towards contralateral space (Figure 2). Biases were not attributable to primary sensory deficits, suggesting higher‐order disturbances.

Proprioceptive biases were attenuated by vision, especially in PCA and tAD groups (increased vision*target position interaction vs Controls: PCA p=.004; tAD p<.001). However, in the PCA group, biases arising from targets in contralateral space were evident even with vision (Figure 2i), indicating altered visual‐proprioceptive interactions.

**Conclusion:**

Perceptual distortions may emerge from altered integration of visual and sensory information across AD phenotypes, especially in atypical presentations. Current digital vision tests offer promise to discriminate PCA from common eye conditions. Adapted tasks are available to detect dementia‐related perceptual disturbances across clinical services and research settings.